# Bioinformatics analysis of the prognosis and biological significance of VCAN in gastric cancer

**DOI:** 10.1002/iid3.414

**Published:** 2021-02-25

**Authors:** Xiao‐yan Huang, Jin‐jian Liu, Xiong Liu, Yao‐hui Wang, Wei Xiang

**Affiliations:** ^1^ Department of Gastric Surgery Fujian Medical University Union Hospital Fuzhou Fujian China; ^2^ Department of Reproduction Luohu District People's Hospital Shenzhen Guangdong China; ^3^ Department of Plastic Surgery Ji 'an Second People's Hospital Ji 'an Jiangxi China; ^4^ Department of Gastrointestinal Surgery Shenzhen People's Hospital Shenzhen Guangdong China; ^5^ Department of Neurosurgery Shenzhen People's Hospital Shenzhen Guangdong China

**Keywords:** gastric cancer, immune infiltration, tumor microenvironment, VCAN

## Abstract

Gastric cancer (GC) is one of the most common cancers in the world, and the tumor microenvironment (TME) plays a vital role in the occurrence and development of GC. In this study, we obtained differential expressed genes in GC tissues from The Cancer Genome Atlas. After ESTIMATE and weighted correlation network analysis, we obtained differentially expressed genes (DEGs). With further screening DEGs of immune infiltration and then through Kaplan‐Meier survival analysis, least absolute shrinkage and selection operator regression analysis and COX analysis, we found that VCAN was a gene positively correlated with high immune infiltration and poor prognosis of patients in GC. In addition, we selected a Gene Expression Omnibus dataset (GSE84433) to verify the effect of VCAN on the patient's prognosis, and analyzed the value of VCAN in immunotherapy through TIMER database and TISIDB. In conclusion, we hold the view that VCAN may affect the development of GC by regulating the TME, which may act as a potential therapeutic target for GC.

## Graphical abstract

VCAN is not only associated with poor prognosis of GASTRIC cancer but also associated with immune infiltration, and maybe a key molecule in immunotherapy for gastric cancer.

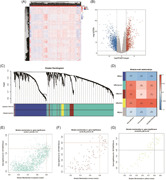

## 1 INTRODUCTION

Gastric cancer (GC) is one of the most common cancers in the worldwide, especially in East Asia.^1^ Studies have shown that more than 70% of cases (677,000) is found in developing countries, and about half occurred in East Asia.^2^ Because the symptoms of early GC are not obvious, and advanced GC is often accompanied with metastasis,^3^ the five‐year survival rate of GC is still very low.^4^ Therefore, the identification of specific molecular markers and therapeutic targets is essential for the diagnosis and treatment to GC patients.

VCAN belongs to the Aggrecan/Versican proteoglycan family.^5^ The VCAN protein is a kind of chondroitin sulfate proteoglycan, which is the important component of extracellular matrix.^5^ Studies have found that VCAN domain interact with a variety of molecules to regulate the adhesion, proliferation, apoptosis, migration, and angiogenesis of multiple cells.^6^ Although VCAN has been studied in several cancers, its research in GC is still unclear.^7^

A counting number of studies have shown that the tumor microenvironment (TME) has an important impact on tumor development.^8^ The microenvironment of tumor tissue includes tumor cells, tumor‐related normal epithelial cells, stromal cells, immune cells, and vascular cells.^9^ Infiltrating stromal cells and immune cells are the main components of normal cells in microenvironment of tumor tissue, which not only interfere with tumor signals in molecular research, but also play an important role in tumor biology.^9^ Besides, tumor‐infiltrating immune cells in the TME are regarded as effective biomarkers of tumor therapy.^10^ In GC, immune cell infiltration is identified as an independent prognostic factor and helps to predict the overall survival rate of GC patients after chemotherapy.^11^ For example, increased CD8 infiltration is related to progression‐free survival and impaired overall survival. GC patients with more CD8 T cell also have higher PD‐L1 expression.^12^ ESTIMATE is a tool to predict tumor purity, stromal cells and immune cells infiltrated in tumor tissues through the gene expression data, and it generates stromal score, immune score, and ESTIMATEScore based on a single‐sample gene set enrichment analysis (ssGSEA).^9^ Therefore, we supposed to screen the genes relate to TME by ESTIMATE.

In this study, we obtained differentially expressed genes (DEGs) in GC tissue of The Cancer Genome Atlas (TCGA). For further screening the differential genes related to the tumor microenvironment in GC, we used weighted correlation network analysis (WGCNA) to screen out 43 differential genes according to ESTIMATE score. It is also because immune infiltration can predict the effect of treatment in the tumor microenvironment.^10^ We tested the relationship between 43 gene expression and immune cell infiltration. It was found that the expression levels of 22 genes had statistically significant difference between the high and low immune infiltration groups. Subsequently, through Kaplan–Meier survival analysis, least absolute shrinkage and selection operator (LASSO) regression analysis and COX analysis, we found that VCAN may be not only an immune‐differential gene, but also an independent risk factor for poor prognosis in GC patients. With analyzing the data from Gene Expression Omnibus (GEO) dataset (GSE84433), TIMER database and TISIDB, we confirmed that VCAN is associated with immune infiltration and prognosis of GC patients. Therefore, we believed that VCAN had the potential to role as a biomarker for predicting the prognosis of GC, and VCAN may affect the TME of GC by regulating the immune infiltration of GC cells.

## 2 MATERIALS AND METHODS

### 2.1 Public database collection

We obtained the high‐throughput sequencing data of GC from TCGA‐STAD.^13^ As |logFC| > 1.5, *p* < .01 were defined as standard of DEGs, we acquired DEGs through limma R package.^14^ Besides, GEO data set (GSE84433) were selected to verified the clinical value of VCAN. Furthermore, the immune public database, TIMER database (https://cistrome.shinyapps.io/timer) and TISIDB (http://cis.hku.hk/TISID), were used for further investigating the sense of VCAN in the immune infiltration of GC and the corresponding immunotherapy. KM‐plotter database (http://www.kmplot.com) was an online database to plot Kaplan‐Meier survival curve from the clinical data of TCGA and GEO.

### 2.2 Single sample gene set enrichment analyses

ssGSEA was an extension of gene set enrichment analyses (GSEA) method, which was mainly designed for the single sample that could not perform GSEA.^15^ For further investigating the immune infiltration in GC, we performed ssGSEA analysis to the expression data from TCGA‐STAD and GEO according to 29 immune genesets^16^ through GSVA R package. Then, according to the result of ssGSEA analysis, we divided samples into high/low immune infiltration groups by clustering.

### 2.3 CIBERSORT

CIBERSORT^17^ was a method to calculate the proportion of 22 immune cells in each sample based on the gene expression data.

### 2.4 ImmuneScore, StromalScore, and ESTIMATEScore

ESTIMATE was implemented by running the ESTIMATE Package in R Language,^18^ which was performed to quantify the immune infiltration and tumor purity in each sample. ImmuneScore, StromalScore, and ESTIMATEScore separately represented the level of immune infiltration, the level of stromal cells infiltration, and the purity of tumors respectively.

### 2.5 Weighted correlation network analysis

WGCNA was used for the construction of co‐expressed network, which divided genes with similar functions into the same module.^19^ As 1997 differentially expressed genes were obtained from TCGA‐STAD, WGCNA analysis were used to acquire module genes by analyzing the score from ESTIMATE.

### 2.6 Kyoto Encyclopedia of Genes and Genomes and Gene Ontology enrichment analysis

Clusterprofiler R package^20^ was applied for Gene Ontology (GO) enrichment analysis, which included molecular function and biological process as well as cellular component of DEGs,^21^ and Kyoto Encyclopedia of Genes and Genomes (KEGG) enrichment analysis. *p* < .05 was defined as significant.

### 2.7 Protein–protein interaction

For further analyzing the relationship among proteins, STRING v11 database^22^ was applied to construct protein–protein interaction (PPI) network.

### 2.8 COX univariate analysis and least absolute shrinkage and selection operator regression analysis

COX univariate analysis was used to determine the risk factors for GC patient prognosis. Besides, LASSO regression analysis, an estimation for processing data with complex collinearity, was performed to screen the prognosis‐related genes. And COX univariate analysis and LASSO regression analysis was performed by R Language.

### 2.9 Survival analysis

Overall survival in GC patients were investigated by Kaplan–Meier analysis, which was conduct by survival R package.

### 2.10 Wound healing assay

GC cell AGS was plated in a six‐well plate with same number. When the cell confluence in the culture dish reached 100%, draw straight lines in the petri dish, then placed it in an incubator at 37°C for 24 h with 5% carbon dioxide in humid air and photograph in the third day at the marked position.

### 2.11 Transwell

GC cell AGS was digested with microprotease, 2 × 10^5^ cells were implanted in each transwell test tube, and 200ul rppi‐1640 containing 10% fetal bovine serum was added. The test tube was then placed in a polycarbonate gel 24‐well plate (BD Biosciences), and rotated at 37°C after 24 h, and the invasion was photographed and observed.

### 2.12 Western blot analysis

The tissues and cells were decomposed with radioimmunoprecipitation assay buffer (Beyotime), separated by 10% sodium dodecyl sulfate polyacrylamide gel electrophoresis, and then transferred to a polyvinyl fluoride membrane (EMD Millipore). Transition to primary antibody and primary antibody reducing solution (Thermo Fisher Scientific). After washing with Tris‐buffered saline with Tween 20 (TBST), the membrane and primary antibody were separated at room temperature for 1 h, and then washed with TBST three times.

### 2.13 Statistical analyses

The significance Statistical difference was determined by *t* test, which was performed by R. As *p* < .05, the difference was considered as statistically significant.

## 3 RESULT

### 3.1 ESTIMATEScore in TCGA‐STAD and WGCNA analysis of DEGs

**Figure 1:**
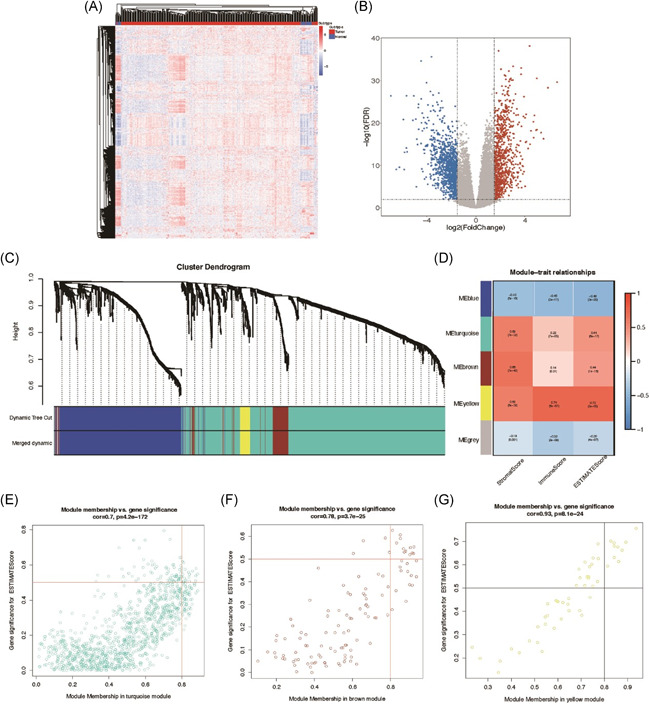
ESTIMATEScore in The Cancer Genome Atlas (TCGA)‐STAD and weighted correlation network analysis (WGCNA) of DEGs. (A) Hierarchical clustering analysis of differentially expressed genes in gastric cancer tissues and adjacent tissues from TCGA database. (B) The Volcano Plots showed the genes with low expression (blue) and high expression (red) in gastric cancer tissues. The screening condition was |logFC| > 1.5, *p* < .01. (C) Cluster Dendrogram was used for the enrichment of gene modules related to immune infiltration, stromal cell infiltration, and ESTIMATEScore. (D) The correlation between each module and immune cell, stromal cell infiltration, and ESTIMATEScore was visualized by Module‐trait relationship. (E–G) The genes in three modules (turquoise, brown, yellow) were screened with ESTIMATEScore > 0.5, module membership >0.8. DEG, differentially expressed gene

In this study, we acquired the expression data of STAD from TCGA database. With the filtering criteria (|logFC| > 1.5, *p* < .01), we obtained 1997 DEGs including 875 upregulated genes and 1127 downregulated genes (Figure 1A,B). To investigate the relationship between GC and tumor microenvironment, we performed WGCNA analysis to 1997 DEGs (Figure 1C) and separately acquired stromal score, immune score, ESTIMATEScore (Figure 1D). It was obviously that modules in color turquoise, brown and yellow were significantly correlated with ESTIMATEScore. As ESTIMATEScore was the sum of ImmuneScore and StromalScore (26), we found that hub genes in these 3 modules had significant correlation with immune cells and Stromal cells infiltration in STAD. After screening (Gene significance for ESTIMATEScore > 0.5, module membership >0.8), 43 hub genes were revealed in 3 modules (Figure 1E‐G). Then, after correlation analysis and cluster were employed to investigate 43 hub genes, we found that genes in the module with same color had high correlation indeed (Figure S1A), which demonstrated that results of our WGCNA analysis were significant. In addition, we performed enrichment analysis to these genes via KEGG and GO, which discovered that these genes had function in extracellular and Osteoclast differentiation (Figure S1B–F). Furthermore, STRING database was used to establish PPI network of these hub genes (Figure S2).

### 3.2 VCAN is associated with immune infiltration and prognosis of GC patients

**Figure 2:**
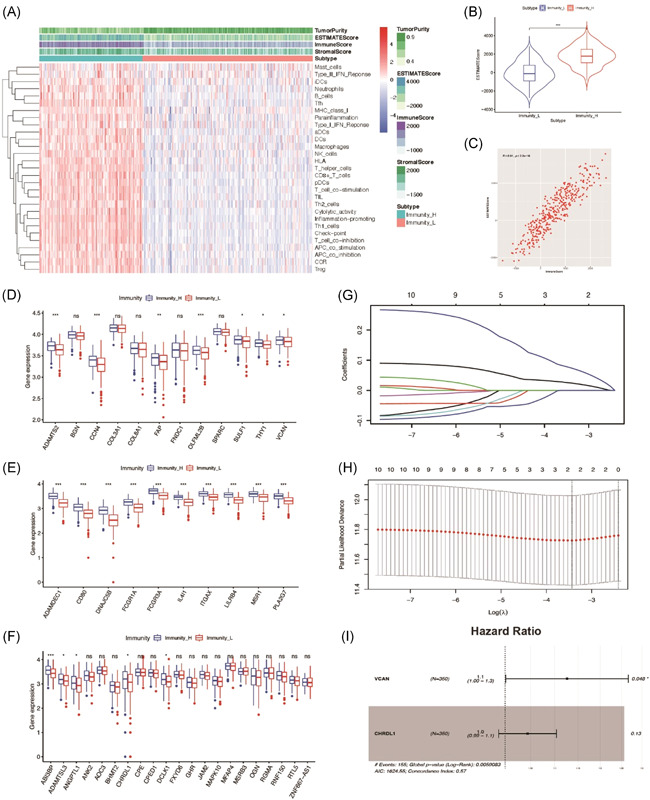
VCAN is associated with immune infiltration and prognosis of GC patients. (A) Immune infiltration heatmap showed the relationship between immune infiltration with tumor purity, stromal score, immune score, and ESTIMATEScore. (B, C) The violin figure and scatter diagram showed that the tumor immune infiltration was positively correlated with ESTIMATEscore. (D–F) Genes expression was significantly different between high and low immune infiltration group. (G, H) LASSO regression analysis was used to identify genes involved in survival. (I) COX analysis was used to further screen for genes that affect survival. GC, gastric cancer; LASSO, least absolute shrinkage and selection operator

As immune infiltration can predict the effect of treatment in the tumor microenvironment,^10^ we further verified the relationship between ESTIMATEscore and immune infiltration. According to several subsets of immune genes, we performed ssGSEA and divided 375 tumor samples in STAD into 2 groups (high immune infiltration vs. low immune infiltration) by cluster analysis. Furthermore, we verified our different immune infiltration groups via the ESTIMATEscore. As shown in Figure 2A‐C, high immune infiltration group had significant higher ESTIMATEscore when comparing with low immune infiltration group, which indicated that ESTIMATEscore could significantly evaluate immune infiltration. To further screen genes regulated immune infiltration, we compared the expression of 43 hub genes from 3 modules between high/low immune infiltration groups, and 22 hub genes had significant different expression (Figure 2D‐F). Besides, we performed Kaplan‐Meier survival analysis to the 22 hub genes via KM‐plotter, whose result showed that 10 hub genes were significant in survival analysis according to the survival data of TCGA‐STAD (Figure S3A–J). According to the overall survival outcome of 10 genes, LASSO regression analysis was used to filter genes and 2 genes (VCAN, CHRDL1) were remained (Figure 2G,H). Furthermore, COX analysis was performed for further verification, which demonstrated that *p* value of VCAN was significant (Figure 2I). Therefore, we prefer VCAN and consider that VCAN is associated with not only the immune infiltration but also prognosis of GC patients.

### 3.3 VCAN act as a tumor promoter in gastric cancer

**Figure 3:**
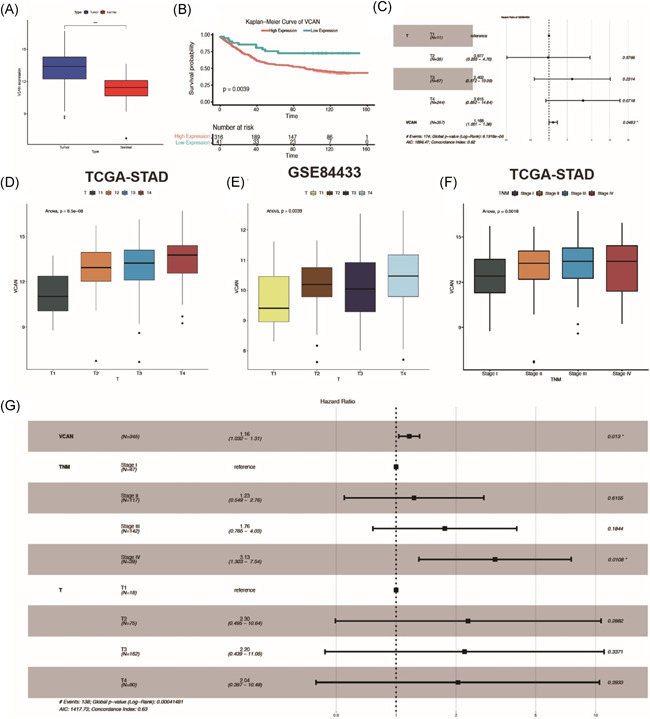
VCAN act as a tumor promoter in gastric cancer. (A) The expression of VCAN in gastric cancer and adjacent tissues. (B) Kaplan–Meier analysis showed that the higher the expression level of VCAN, the worse the prognosis of gastric cancer patients. (C) COX analysis showed that T stage and VCAN were both poor prognostic factors. (D, E) The box diagram showed the relationship between VCAN expression and T staging in TCGA and GEO database, respectively. (F) The box diagram showed the relationship between VCAN expression and TNM stage. (G) COX analysis showed that VCAN and T staging were associated with poor prognosis. GEO, Gene Expression Omnibus; TCGA, The Cancer Genome Atlas

To investigated the clinical value of VCAN, we obtained genetic data of GC expression in TCGA‐STAD and found that VCAN was highly expressed in GC tissues relative to adjacent tissues (Figure 3A). As high VCAN could lead to poor overall survival prognosis in STAD, we further analyzed it in the GSE84433 and found that VCAN act as a tumor promoter in GC (Figure 3B). For further research in clinical, we selected other prognosis features from GSE84433 and found that T stages was significantly correlated with VCAN expression (Table 1, Figure 3C,D). For further convince, we chose data from TCGA‐STAD to investigate the impact of VCAN to other clinical features (Table 2), which demonstrated that T stages and TNM stages (Figure 3E,F) were significantly correlated with VCAN expression. As COX analysis performed, Stage IV and VCAN both had significant *p* value (Figure 3G), which indicated that both Stage IV and VCAN expression were significant tumor promoter in GC.

**Table 1:** Correlation between VCAN and clinical features in GSE84433

	**VCAN**		
	**High**	**Low**	***p* Value**
Age			.596
≥60	180	21	
<60	136	20	
Gender			.6461
Male	216	26	
Female	100	15	
T stage			**.0018**
T1	7	4	
T2	31	4	
T3	53	14	
T4	225	19	
N stage			.3596
N0	59	12	
N1	137	18	
N2	91	8	
N3	29	3	

*Note*: Bold values indicate that the difference is statistically significant, *p* < .05.

**Table 2:** Correlation between VCAN and clinical features

	**VCAN**		
	**High**	**Low**	***p* Value**
T stage			**.0008**
T1	0	18	
T2	13	62	
T3	37	125	
T4	33	57	
N stage			.1184
N0	19	89	
N1	25	71	
N2	17	58	
N3	22	44	
M stage			.0651
M0	73	248	
M1	10	14	
TNM stage			**.0024**
Stage I	3	44	
Stage II	27	90	
Stage III	38	104	
Stage IV	15	24	
Disease type			.4159
Adenomas and adenocarcinomas	74	243	
Cystic, mucinous, and serous neoplasms	9	19	

*Note*: Bold values indicate that the difference is statistically significant, *p* < .05.

### 3.4 Relationship between VCAN and immune infiltration

**Figure 4:**
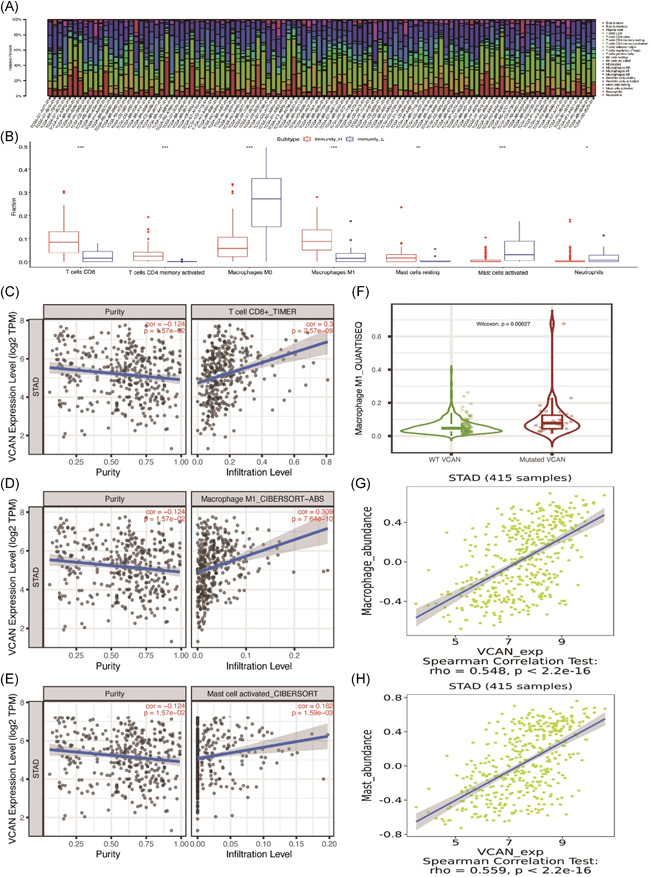
Relationship between VCAN and immune infiltration. (A) CIBERSORT was used to analyze the immune infiltration of 22 immune cells. (B) 7 immune cells (T cells CD8+, T cells CD4 memory activated, macrophages M0, macrophage M1, mast cells resting, mast cells activated, neutrophils) were supposed to have different levels of infiltration between high/low immunity group. (C–E) According to the TIMER database, 3 immune cells (T cell CD8+, macrophages M1, mast cell activated) were suggested to have a significant correlation after the adjustment of purity. (F) Macrophage M1 had significant high expression in mutated VCAN group, which indicated that mutation VCAN may induce the high immune infiltration of macrophage M1. (G, H) According the TISIDB, macrophage and mast cells were supposed to have significant correlation with VCAN expression

To verify the correlation between VCAN and immune infiltration, we selected another GEO dataset (GSE84433) for further investigation. Besides, after we performed ssGSEA analysis and evaluated ESTIMATEScore, the relationship between immune infiltration and ESTIMATEscore was verified (Figure S4A–C). It was obvious that ESTIMATEScore had significant different expression between high/low immune infiltration groups. Therefore, we further estimated expression of VCAN in different groups of GSE84433 and revealed that VCAN had high expression in high immune infiltration group, which indicated that VCAN expression was positively correlated with immune infiltration level (Figure S4C. Then we transformed gene expression data of TCGA‐STAD into the matrix of immune cells in GC samples via the CIBERSORT, which offered us the proportion of immune cells in each sample (Figure 4A). Furthermore, as samples in TCGA‐STAD were divided into high or low immune infiltration groups, we estimated the different expression of each immune cell and found that 7 immune cells (T cells CD8, T cells CD4 memory activated, macrophages M0, macrophages M1, Mast cells resting, Mast cells activated, Neutrophils) had significant different expression between two groups (Figure 4B). As VCAN had high expression in high immune infiltration group, we verified the relationship between VCAN expression and immune infiltration level in TIMER database and found that only three immune cells (T cell CD8+, macrophage M1, mast cell activated) had corresponding trend (Figure 4C‐E). Besides, we also focused on the mutation of VCAN through TIMER database, which demonstrated that mutated VCAN was positively correlated with high immune infiltration of macrophage M1 in GC (Figure 4F). Afterwards, we expanded our research by using TISIDB to investigate the correlation between VCAN expression and immune cells, which demonstrated that macrophage M1 (*R* = 0.548, *p* < 2.2 × 10^−16^) and mast (*R* = 0.548, *p* < 2.2 × 10^−16^) were significantly correlated with VCAN expression (Figure 4G‐H). According to the different analysis tools (TIMER, TISIDB), we held the view that VCAN was significantly correlated with the immune infiltration of several immune cells indeed.

### 3.5 The value of VCAN in immune therapy

**Figure 5:**
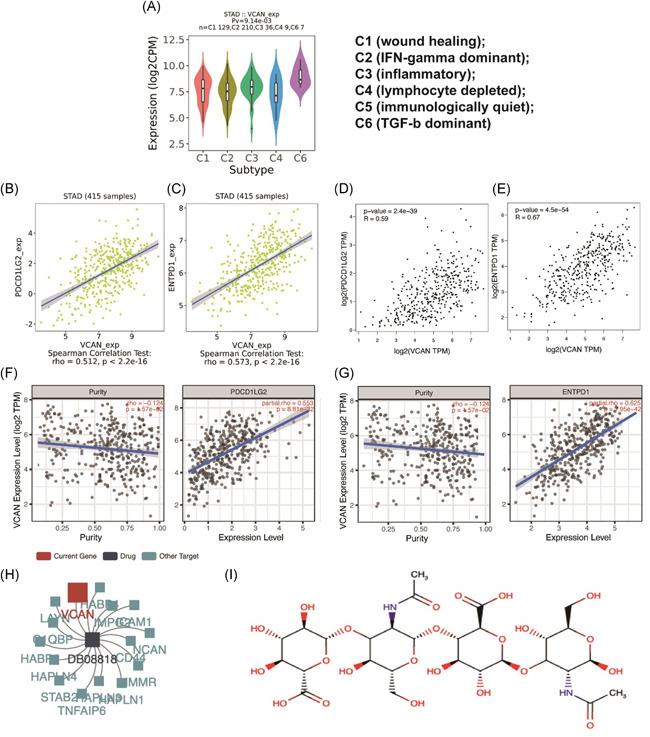
The value of VCAN in immune therapy. (A) In TISIDB, VCAN had the most high expression in TGF‐b dominant type of gastric cancer significantly (*p* = 9.14 × 10^−3^). (B, C) After analyzing in TISIDB, VCAN was significantly correlated with two immune biomarkers in immunotherapy (PDCD1LG2, ENTPD1). (D, E) The correlation between two biomarkers and VCAN were verified through GEPIA which demonstrated that VCAN was significantly correlated with two genes indeed. (F, G) According to the result from TIMER database, VCAN had a significant correlation with two genes after the adjustment of tumor purity. (H, I) VCAN was found to be the target of the hyaluronic acid. IFN, interferon; TGF, tumor growth factor

As VCAN was a significant immune infiltration biomarker, we were curious of the function of VCAN in immune therapy. Therefore, we performed further data mining via TISIDB. As GC was divided into five immune subtypes in TISIDB, we found that VCAN had highest expression in TGF‐B subtype, which indicated that VCAN may role as an immune factor in this GC subtype (Figure 5A). Then, for investigating its function in immune molecular therapy, we investigated the correlation between VCAN and each immune regulator, which included immunostimulator, immunoinhibitor and MHC molecular (Figure S5–7). After screening (|R| > 0.5), PDCD1LG2 as well as ENTPD1 were regarded as molecular with strong correlation (Figure 5B,C). Besides, to verify the results from TISIDB, we also investigated their correlation from GEPIA and TIMER for confirm, which both demonstrated that VCAN had strong significant correlation with these two genes (PDCD1LG2, ENTPD1) (Figure 5D‐G). Furthermore, we also revealed that VCAN role as a target site of hyaluronic acid from TISIDB, whose data was originated from Drugbank database. As hyaluronic acid was reported to participate in immune therapy in several articles, a new direction of investigating the impact from VCAN to immune infiltration in GC was provided.

### 3.6 Effect of VCAN on function of GC cells and CD44

**Figure 6:**
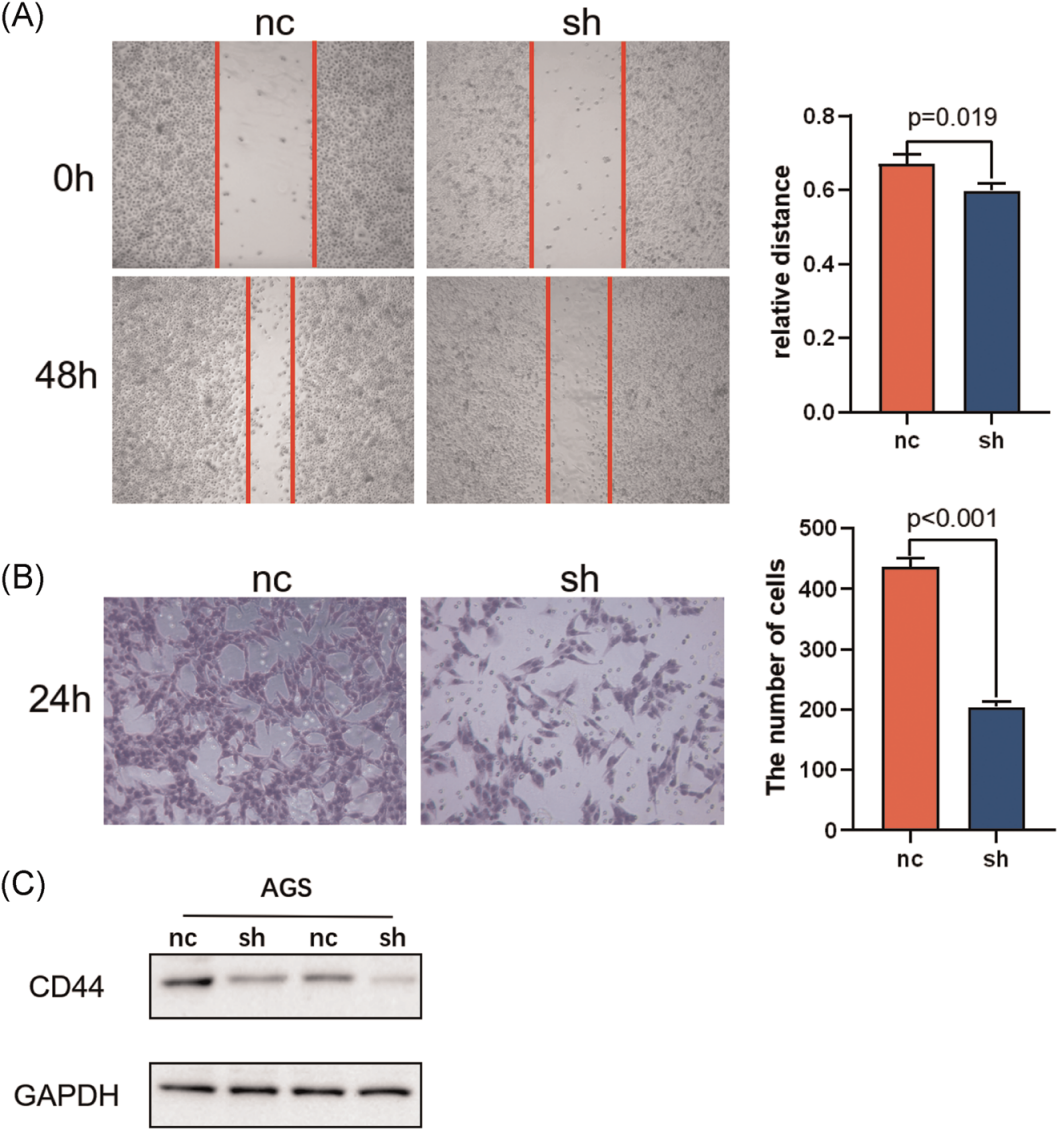
(A) The effect of VCAN on AGS migration ability of gastric cancer cells was detected by Wound healing assay. (B) Transwell detected the effect of VCAN on the invasion ability of AGS in gastric cancer cells. (C) Western blot detected the effect of VCAN on the expression of immune protein CD44. GAPDH, glyceraldehyde 3‐phosphate dehydrogenase

To verify the reliability of the data analysis, we knocked down VCAN in AGS of GC cells, and found that the migration ability of AGS of GC cells was weakened (Figure 6A). In addition, through Transwell invasion experiment, we found that the invasion ability of AGS of GC cells was decreased after VCAN was knocked down (Figure 6B), indicating that VCAN can promote the invasion and migration of GC cell. In addition, we found by Western blot that the expression of immune‐related protein CD44 was decreased after VCAN was knocked down (Figure 6C), so we believed that VCAN was immune‐related in GC.

## 4 DISCUSSION

In this study, we tried to find genes affecting GC TME, especially immune infiltration, from TCGA, which could also act as biomarkers for the diagnosis, treatment and regulating prognosis of GC. Therefore, we found that VCAN was not only related with tumor immune infiltration, but also an independent factor for the prognosis of GC patients.

The TME referred to the internal and external environment where tumors occurred, grew, metastasized and the location of tumor cells. It had influence to the development of cancer through not only tumor cells, but also nontumor cells and noncellular components.^23^ In tumor tissues, nontumor cells mainly infiltrated stromal cells and immune cells.^9^ Therefore, by evaluating the status of stromal cells as well as immune cells in tumor tissues, ESTIMATE could indirectly transform the TME. ESTIMATE used gene expression data to generate stromal score, immune score and ESTIMATEScore based on ssGSEA analysis.^9^ Besides, ESTIMATEScore represented the comprehensive ratio of ImmuneScore and StromalScore in the TME. Therefore, to find genes related to TME, we acquired genes of different functional modules through WGCNA, and screened 43 genes related to ESTIMATEScore. As infiltrating immune cells played a role in predicting the prognosis of tumor patients in the TME, we further investigated the relationship between the expression of 43 genes and the immune infiltration, which demonstrated that the expression of 22 genes was significantly different in high and low immune infiltration groups. Furthermore, through Kaplan‐Meier survival analysis, we further revealed that the expression of 10 genes is statistically significant to the GC patient's survival. Through LASSO regression analysis and COX analysis, we found that VCAN was an independent risk factor for the prognosis of GC patients. To ensure the sense of the results, we verified it through GEO dataset (GSE84433), TIMER database and TISIDB, and discussed the value of VCAN in immunotherapy. Meanwhile, for exploring the useage of VCAN in clinical therapy, we revealed that VCAN a binding target of hyaluronic acid, which was demonstrated in the DRUGBANK database.^24^ With a simlar structure to hyaluronic acid‐binding proteins in the amino‐terminal domain, the protein of VCAN (versican), with a B‐loop domain, was regarded to performed a binding function towards hyaluronic acid in human.^25^, ^26^, ^27^ Besides, as different functions varied from multiple sizes of hyaluronic acid, we further investigated it to ensure the size of hyaluronic acid targeted by versican. Then, we discovered that reduced amount of high molecular weight hyaluronic acid (high MW HA: 1.5MDa) could lead to low expressed vesican (low MW HA: 40 kDa) in protein level, while change was not found in the fewer expression of low MW HA.^28^ Therefore, we held the view that versican, as a protein production of gene *VCAN*, could target hyaluronic acid with high molecular weight via its structure that resembled hyaluronic acid‐binding proteins, which need further investigation.

VCAN had been found to affect the occurrence and development of a variety of cancers,^6^, ^29^, ^30^ but its research in GC tissue was still unclear. Studies had shown that the high expression of VCAN in GC tissue was an independent risk factor for poor prognosis of patients, which was extremely common in more aggressive tissues.^7^, ^31^ Based on the analysis of the differentially expressed GC genes from TCGA, our study tried to origin from the TME and immune infiltration, which was further combined with the clinical survival prognosis analysis, and found that VCAN affected the prognosis of GC patients which was related to the TME and immune infiltration. Diagnosis and treatment of GC would be offered new guidelines. However, through this bioinformatics analysis only reveal the role of VCAN in immune infiltration and TME of GC. We will perform experiments in vivo and in vitro for further exploring the mechanism of VCAN affecting on GC.

## 5 CONCLUSION

VCAN is highly expressed in GC tissues, whose upregulated expression is correlated with the poor prognosis of GC. In addition, the expression of VCAN is high in the group with high immune infiltration in GC tissue. We believe that VCAN is a new immune‐induced prognostic indicator in GC by regulating the infiltration of several immune cells in GC.

## CONFLICT OF INTERESTS

The authors declare that there is no conflict of interest.

## AUTHOR CONTRIBUTIONS

Xiao‐yan Huang, Jin‐jian Liu, and Xiong Liu collected and analyzing data, drawn in R language, and prepared the manuscript. Yao‐hui Wang and Wei Xiang performed the tests. Xiao‐yan Huang drafted the manuscript. All authors read and approved the final manuscript.

## Supporting information

Supporting information.

Supporting information.

Supporting information.

Supporting information.

Supporting information.

Supporting information.

Supporting information.

## Data Availability

The datasets used in the project are available from the corresponding author. The data that support the findings of this study are openly available in TCGA at http://www.tcga.org/ and GEO at https://www.ncbi.nlm.nih.gov/geo/.
