# Supplementary figures and images for: Bioinformatics analysis of the prognosis and biological significance of VCAN in gastric cancer
Source: Immun Inflamm Dis. 2021 Feb 25;9(2):547–59. doi: 10.1002/iid3.414 (PMC8127546; doi:10.1002/iid3.414)

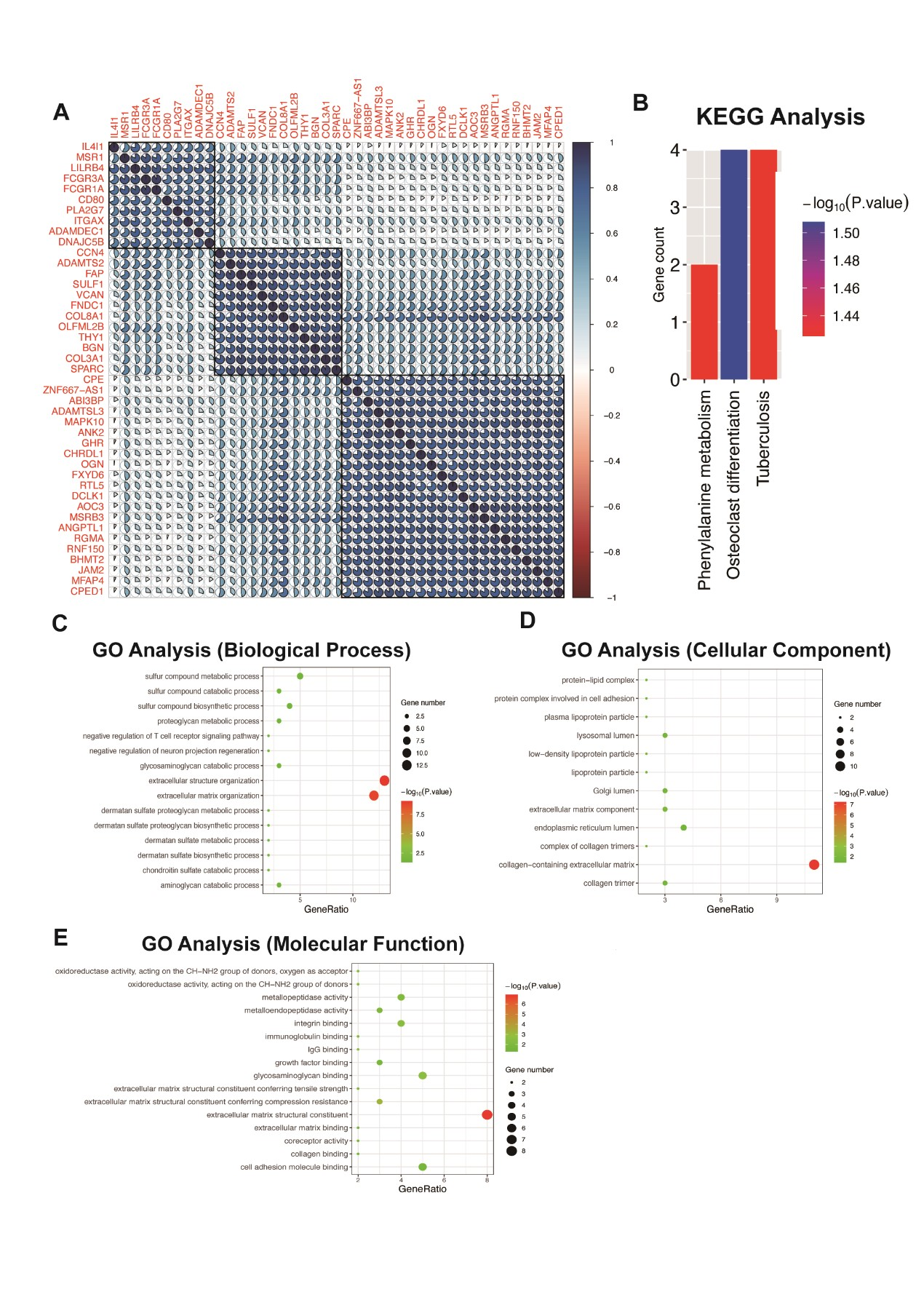

Supplement: Supplementary file 1 — Supporting information. [file IID3-9-547-s001.tif]

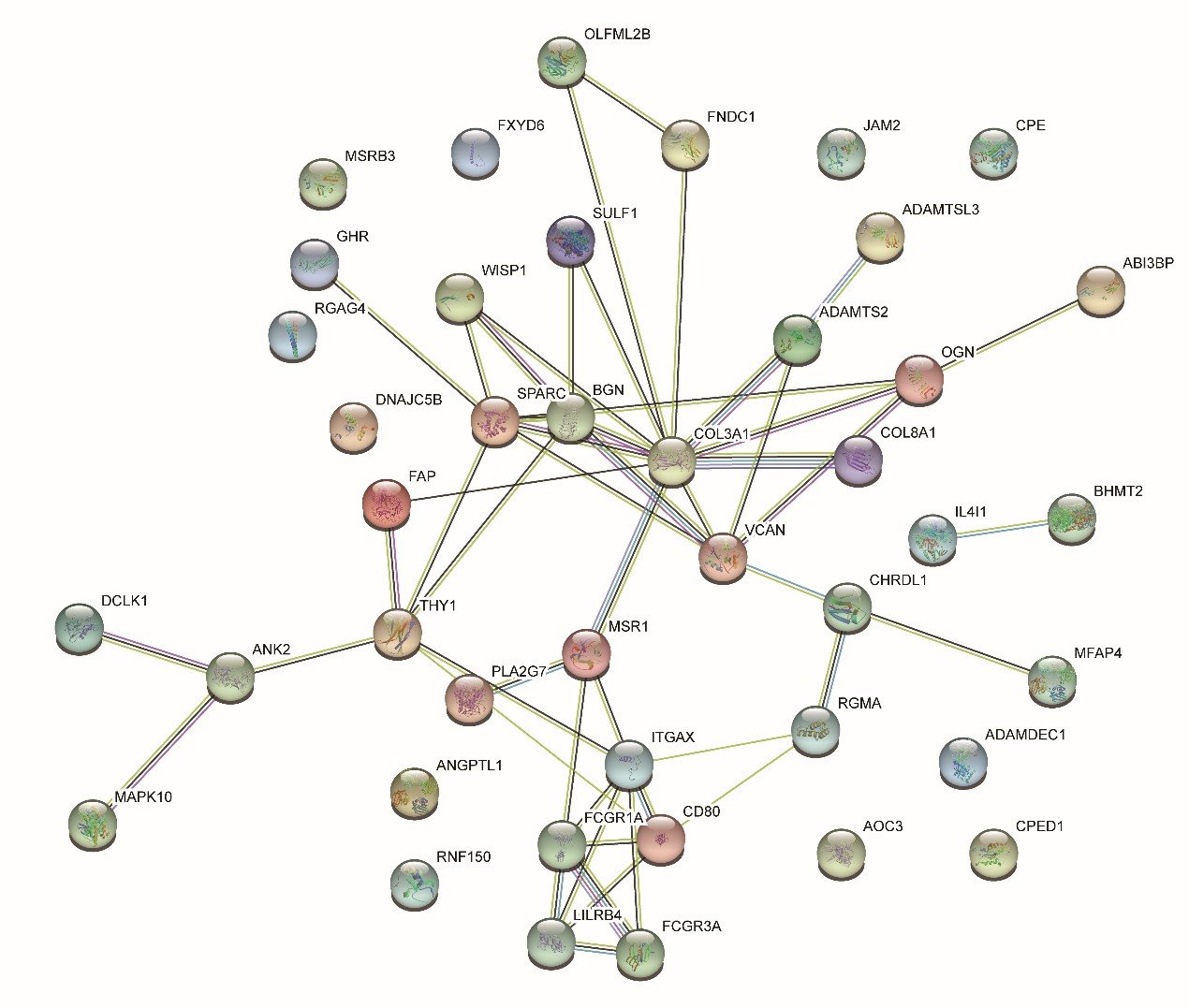

Supplement: Supplementary file 2 — Supporting information. [file IID3-9-547-s002.tif]

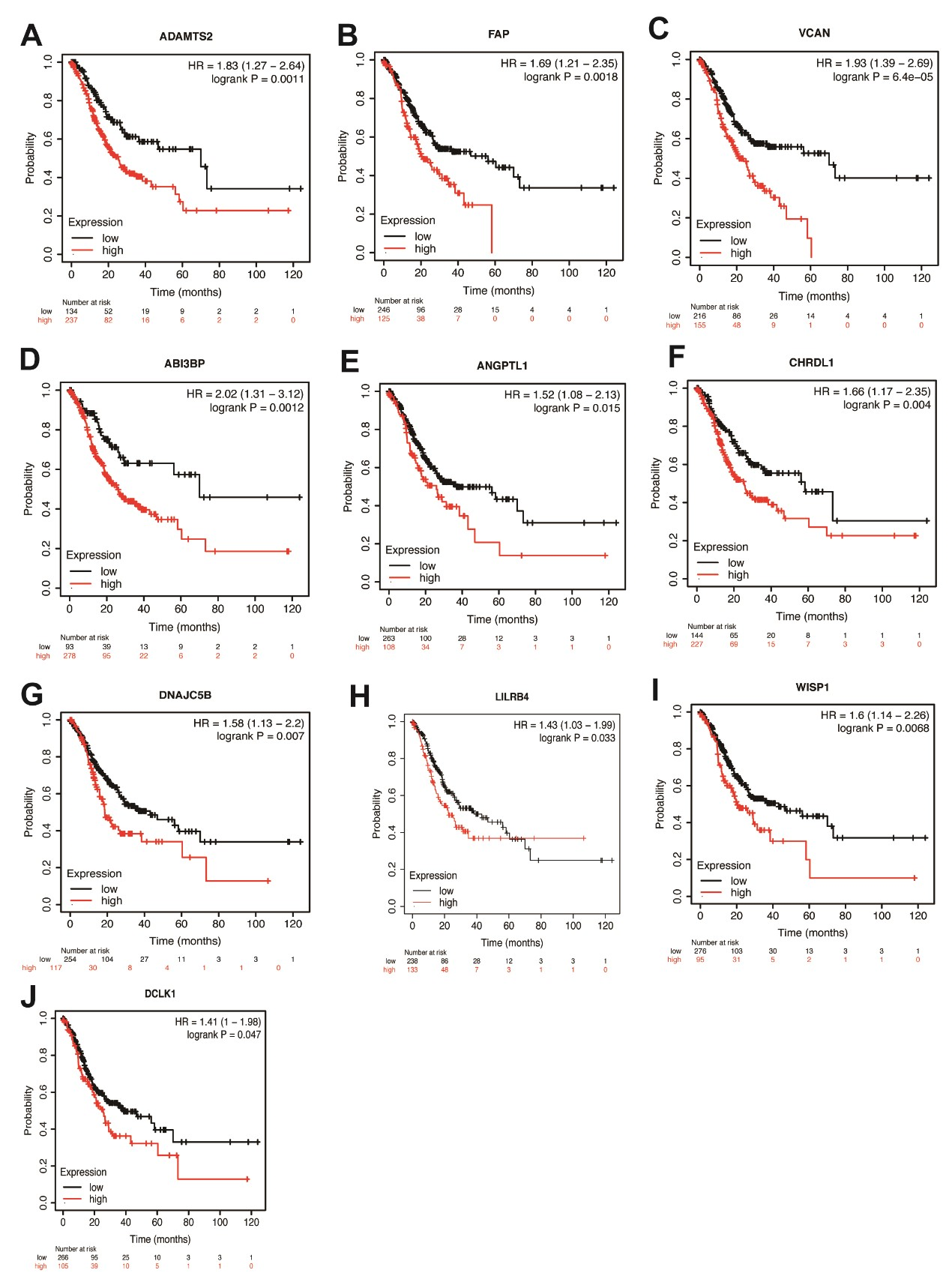

Supplement: Supplementary file 3 — Supporting information. [file IID3-9-547-s005.tif]

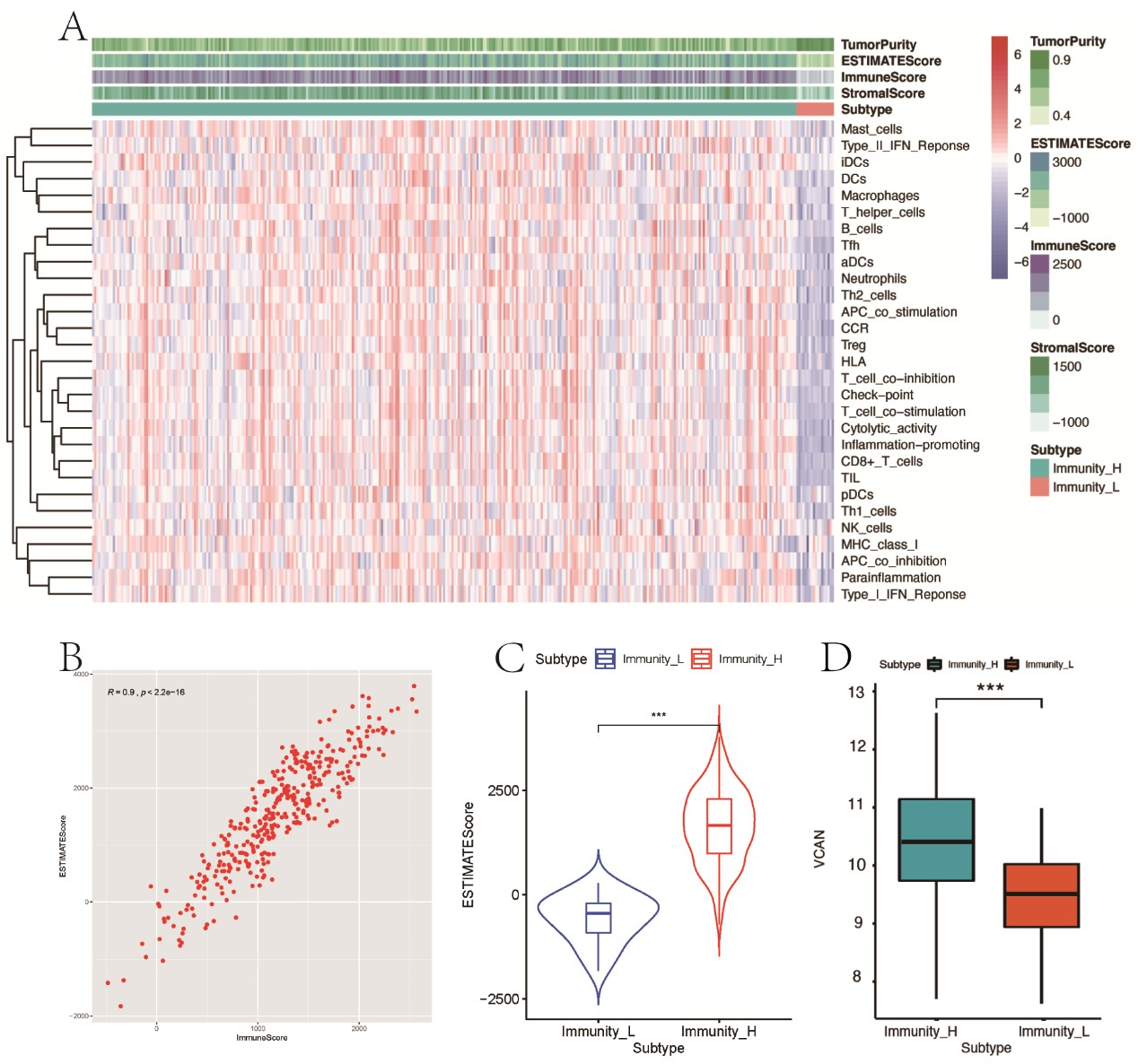

Supplement: Supplementary file 4 — Supporting information. [file IID3-9-547-s006.tif]

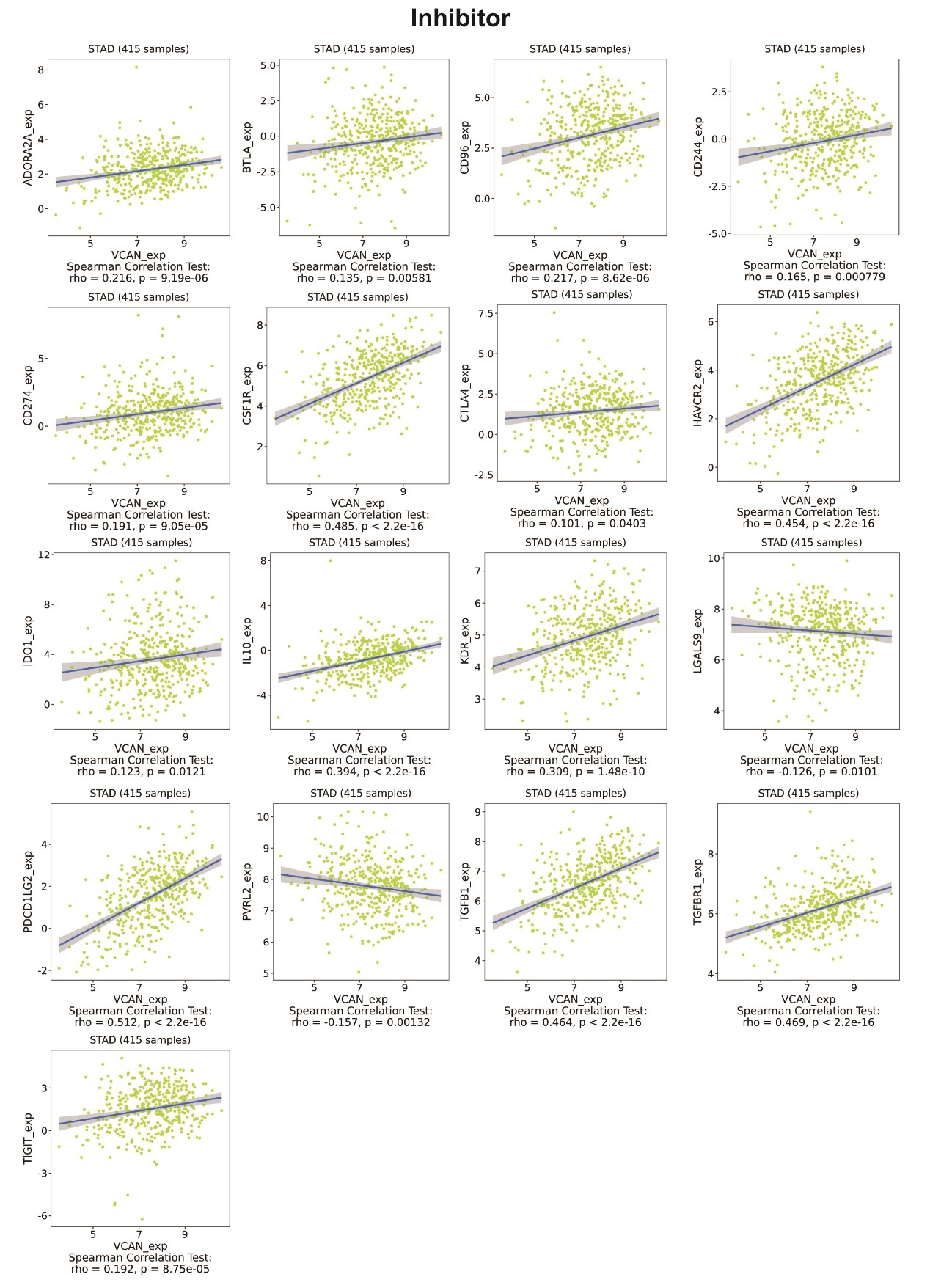

Supplement: Supplementary file 5 — Supporting information. [file IID3-9-547-s004.tif]

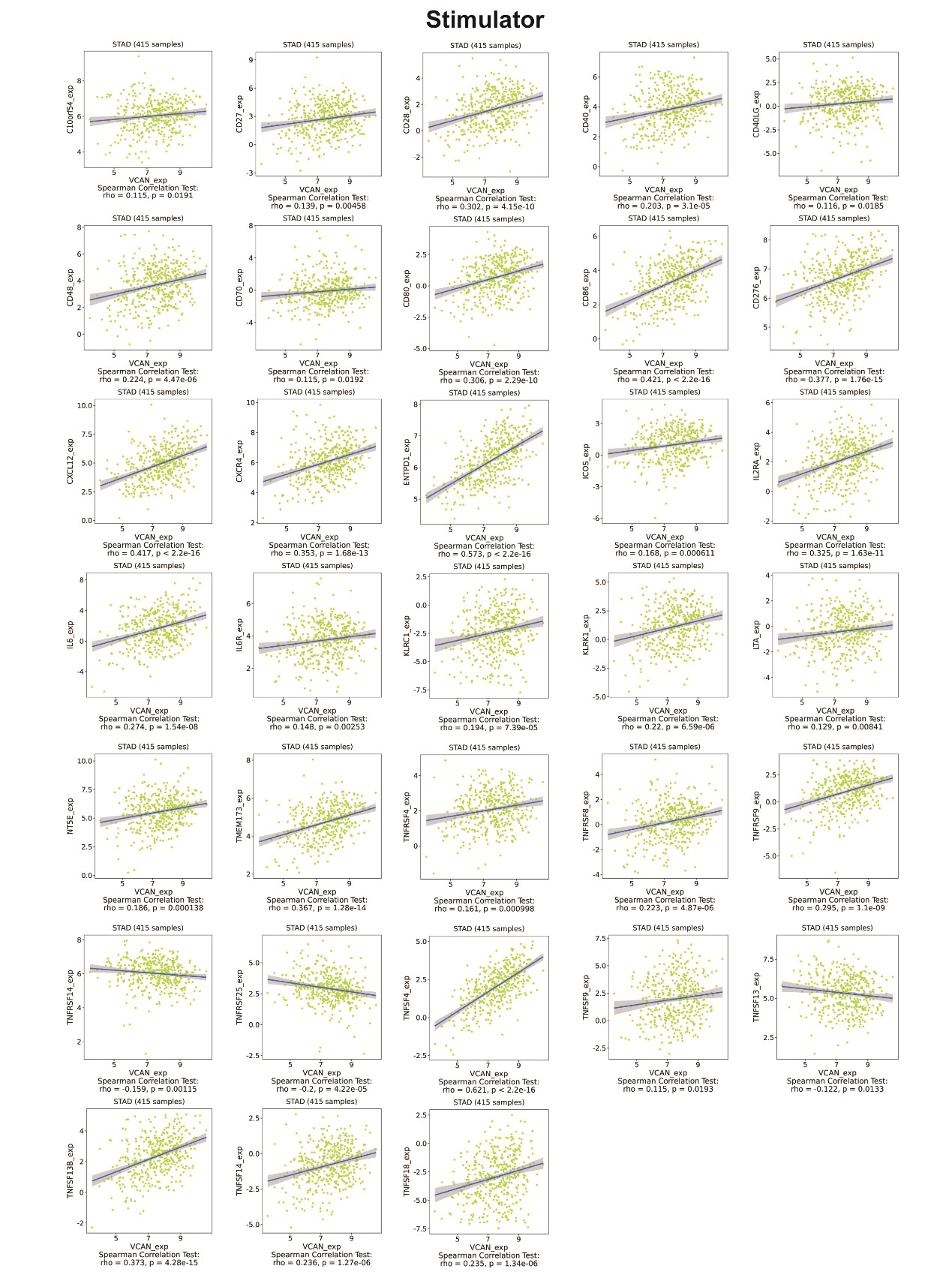

Supplement: Supplementary file 6 — Supporting information. [file IID3-9-547-s003.tif]

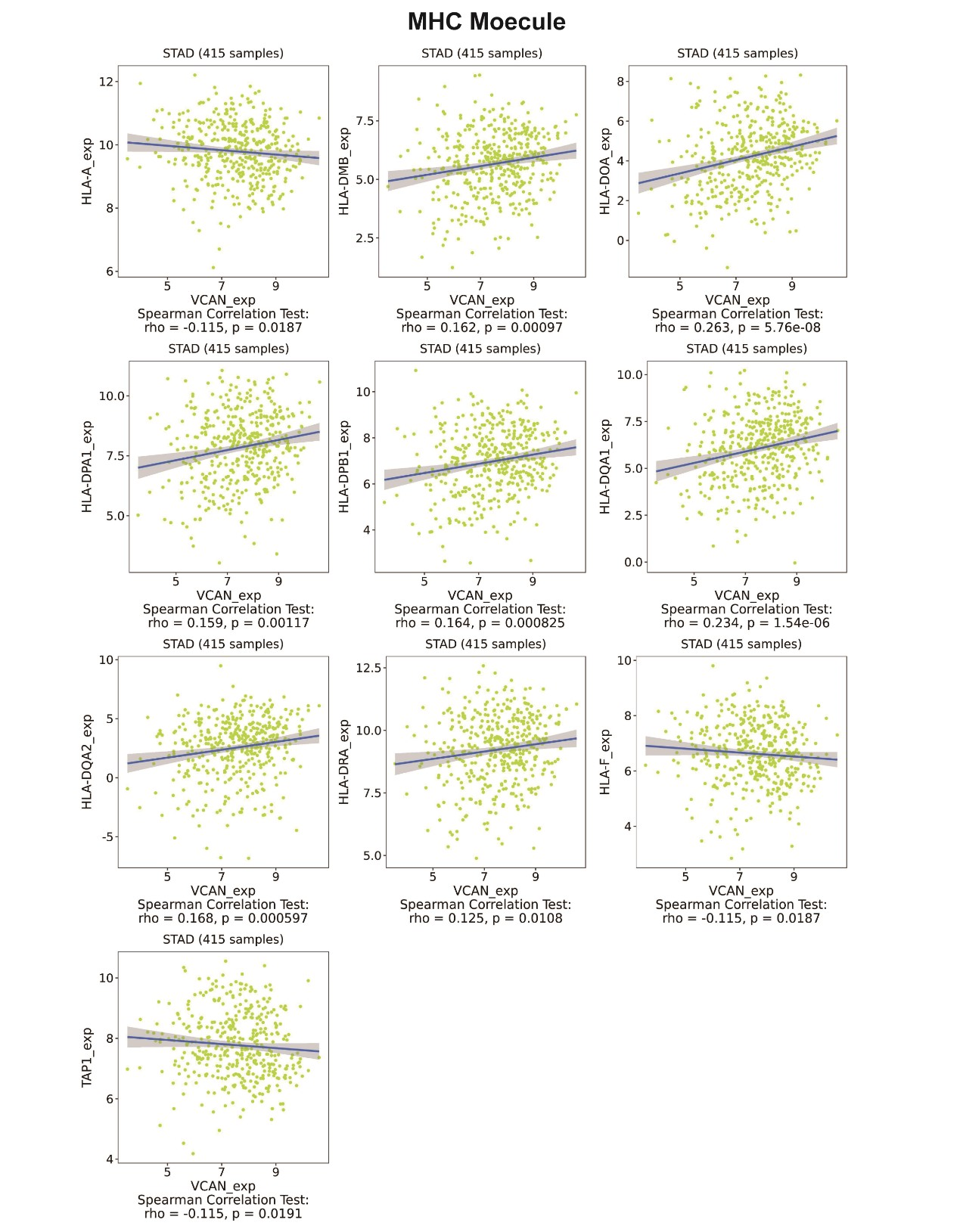

Supplement: Supplementary file 7 — Supporting information. [file IID3-9-547-s007.tif]
